# Gut microbiota–bile acid axis in cardiovascular disease: a spatiotemporal framework for predictive, preventive and personalised medicine

**DOI:** 10.1007/s13167-026-00464-5

**Published:** 2026-08-10

**Authors:** Xiaowen Tian, Haoran Hua, Jie Zhang, Peng Zhao

**Affiliations:** 1 Department of Cardiology, Shandong Provincial Hospital affiliated to Shandong First Medical University, Jinan, Shandong 250021 China; 2 Department of Clinical Nutrition, Shandong Provincial Hospital affiliated to Shandong First Medical University, Jinan, Shandong 250021 China

**Keywords:** Predictive preventive personalised medicine (PPPM / 3PM), Cardiovascular disease, Gut microbiota, Bile acids, Functional phenotyping, Patient stratification

## Abstract

Cardiovascular disease (CVD) retains a substantial residual burden despite control of conventional risk factors, indicating contributions from additional metabolic, inflammatory and host–microbial pathways. The gut microbiota–bile acid axis links diet and environmental exposures to microbial metabolism, enterohepatic signalling and cardiovascular homeostasis. However, current evidence is commonly organised around isolated diseases or molecular mechanisms, with limited attention to when this axis becomes detectable, how it should be clinically interpreted and whether it can guide individualised prevention. In this review, we propose a spatiotemporal framework for the gut microbiota–bile acid axis across the health–suboptimal health–disease continuum within predictive, preventive and personalised medicine (PPPM/3PM). Mechanistic evidence is most developed for atherosclerosis and atherothrombosis, whereas evidence for atrial fibrillation, heart failure, pulmonary hypertension and cerebrovascular outcomes is more heterogeneous and less mature. Microbial bile salt hydrolase activity and downstream bile acid transformations reshape compartment-specific bile acid pools, with potential effects on cholesterol catabolism, inflammatory and barrier responses, vascular homeostasis, platelet activation and myocardial stress. For 3PM, molecular profiles should be interpreted together with multidomain functional phenotypes, including bowel function and gastrointestinal symptoms, hepatobiliary status, metabolic–inflammatory context, and diet and medication exposure. Microbiota-directed interventions should be function-informed rather than selected by taxonomy alone. Translation requires longitudinal multi-omics, standardised multi-compartment bile acid profiling, causal validation in human-relevant models and prospective studies determining whether phenotype-guided strategies add predictive or preventive value beyond established cardiovascular care.

## Introduction

Despite substantial progress in reducing the burden of cardiovascular disease (CVD) through the control of classical risk factors such as dyslipidaemia and hypertension, a considerable residual risk persists, suggesting that additional pathogenic pathways remain insufficiently characterised [1]. Meeting this challenge requires attention to biological layers that connect environmental exposures, diet and host metabolism to cardiovascular phenotypes. Against this background, the diet–gut microbiota–host interaction framework has gained increasing relevance, with structurally diverse microbial metabolites, including bile acids and short-chain fatty acids (SCFAs), recognised as important mediators of host–microbial crosstalk [2, 3]. The gut microbiota is increasingly regarded as a functionally active metabolic and endocrine-like organ, whose composition and activity are shaped by diet and which may exert long-range effects on host physiology, including cardiovascular homeostasis, through a repertoire of bioactive metabolites [4–6].

Within this broader framework, the gut microbiota–bile acid axis represents a particularly relevant signalling interface. Bile acids are not merely end-products of cholesterol catabolism or detergents for lipid absorption; they are also signalling molecules extensively modified by microbial enzymes and decoded by host receptors and metabolite-sensing pathways. Through microbial bile salt hydrolase activity, bile acid biotransformation and enterohepatic signalling, this axis may influence cholesterol metabolism, inflammatory tone, endothelial and barrier integrity, platelet activation and myocardial stress. It therefore provides a biologically plausible link between diet, microbial ecology and heterogeneous cardiovascular phenotypes, especially atherosclerosis, atherothrombosis and cardiometabolic risk [5].

The translational value of this axis, however, cannot be fully captured by an organ-specific or mechanism-centred framework alone. As articulated in the EPMA position framework, predictive, preventive and personalised medicine (PPPM/3PM) shifts the focus from reactive treatment of established disease to the prediction of individual susceptibility, targeted prevention of the health-to-disease transition and personalisation of medical services across the disease continuum [7]. This orientation has already been advocated for cardiovascular medicine [8] and is particularly relevant to suboptimal health, a reversible preclinical state between health and overt disease that provides a key window for predictive diagnostics and targeted prevention [9]. In this setting, the gut microbiota–bile acid axis may be interpreted not only as a mechanistic contributor to CVD, but also as a potentially measurable, modifiable and individualisable system.

Existing reviews have separately summarised the cardiovascular implications of gut microbiota, bile acid metabolism or individual disease phenotypes. What remains insufficiently defined is when along the health–suboptimal health–disease continuum this axis becomes dysregulated, which microbial or bile acid signals can be measured before overt cardiovascular damage, which nodes are suitable for targeted prevention, and how individual microbial and bile acid profiles may guide personalised intervention. In line with a growing body of EPMA work that places gut microbiota-related mechanisms within predictive, preventive and personalised strategies [10], this Review applies a dedicated 3PM perspective to the cardiovascular gut microbiota–bile acid axis.

A PPPM/3PM perspective is most informative when molecular measurements are linked to functional clinical phenotypes. In addition to cardiovascular stage, interpretation of the gut microbiota–bile acid axis should consider bowel function and gastrointestinal symptoms, hepatobiliary status, diet and medication exposure, and the metabolic–inflammatory context. These features are not established cardiovascular predictors; rather, they may help distinguish biologically different subgroups that share superficially similar microbiome or bile acid profiles and may partly explain interindividual variation in intervention response. This multidomain phenotyping layer avoids the assumption that a single microbial taxon, enzyme function or bile acid ratio has uniform clinical meaning across patients.

**Fig. 1:**
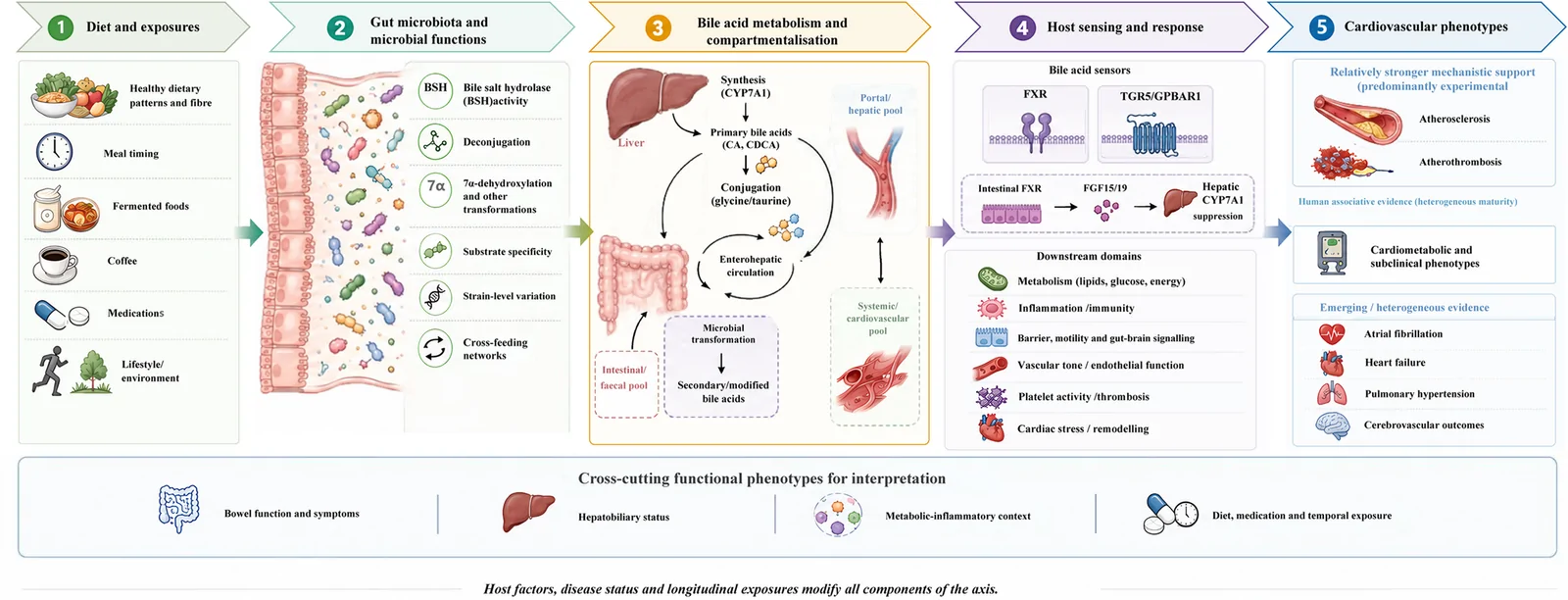
The gut microbiota–bile acid axis: from diet to cardiovascular disease. Dietary and environmental exposures shape microbial bile acid-transforming functions and remodel intestinal/faecal, portal/hepatic and systemic/cardiovascular bile acid pools. These signals are interpreted through FXR–FGF15/19–CYP7A1, TGR5/GPBAR1 and related pathways, with potential effects on metabolic, inflammatory, gastrointestinal, vascular, platelet and myocardial responses. Mechanistic support is relatively stronger for atherosclerosis and atherothrombosis, whereas evidence for other cardiovascular phenotypes is less mature. Cross-cutting functional phenotypes provide interpretive context rather than validated cardiovascular predictors. BSH, bile salt hydrolase; CYP7A1, cholesterol 7α-hydroxylase; FGF15/19, fibroblast growth factor 15/19; FXR, farnesoid X receptor; GPBAR1, G protein-coupled bile acid receptor 1

Here, we propose a spatiotemporal framework for interpreting gut microbiota–bile acid dysregulation in CVD. We distinguish human associative evidence from experimental mechanistic evidence to clarify causal strength and translational readiness. We first map the evidence from early metabolic perturbation and subclinical cardiovascular injury to overt cardiovascular events and multi-organ crosstalk. We then examine major mechanistic hubs, including microbial bile acid biotransformation, FXR–FGF15/19–CYP7A1 signalling, TGR5/GPBAR1-mediated vascular and platelet responses, immune–barrier–endothelial regulation and myocardial stress. Finally, we discuss how microbiome and bile acid measurements may be integrated with gastrointestinal, hepatobiliary, metabolic and digital phenotypes to support predictive diagnostics, function-informed dietary or microbiota-directed prevention, and responder-stratified personalisation of cardiovascular care within the PPPM/3PM framework. Figure 1 summarises the mechanistic framework linking diet and other exposures, microbial bile acid-transforming functions, compartment-specific bile acid pools, host signalling responses and cardiovascular phenotypes.

## Spatiotemporal dysregulation of the gut microbiota–bile acid axis in cardiovascular disease

### Early metabolic perturbations and subclinical cardiovascular injury

Large-scale cross-sectional and cohort studies have reported associations between specific gut microbial features and subclinical markers of atherosclerosis in clinically asymptomatic populations [11]. In the SCAPIS cohort of nearly 9,000 participants, several *Streptococcus* species, including *S. sanguinis* and *S. oralis*, were positively associated with coronary artery calcium scores after adjustment for relevant covariates [11]. Within a PPPM/3PM framework, these findings support prospective evaluation of gut microbial and metabolite profiles as potential sources of candidate risk markers during suboptimal health, but they do not establish temporal precedence or incremental predictive value beyond established cardiovascular assessment [9, 11]. The associated plasma metabolite pattern, including higher secondary bile acids and lower indolepropionic acid, further suggests that microbial features should be investigated together with host metabolic profiles rather than interpreted as isolated taxonomic biomarkers [11].

Comparable associations have been reported in subclinical cardiac dysfunction. In a Hispanic/Latino cohort, left ventricular diastolic dysfunction was associated with reduced abundance of *Intestinimonas massiliensis* and with variation in circulating secondary bile acids [12]. Altered microbial and metabolic profiles, including *Faecalitalea* and *Turicibacter*-related signals and changes in bile acid pathways, have also been reported in hypertensive left ventricular hypertrophy [13]. Taken together, these observations identify candidate microbiome–metabolite signatures associated with subclinical cardiovascular phenotypes, while their temporal sequence, causal relevance and predictive performance require longitudinal validation.

Cardiometabolic risk states, including hypertension, obesity and insulin resistance, have been associated with gut microbial dysbiosis and altered microbial metabolite pathways [14–17]. Bile acid remodelling has also been reported in these contexts, but its direction and consistency vary across populations and experimental models, and the available evidence does not establish a uniform secondary-to-primary bile acid pattern across these heterogeneous risk states [14, 16, 17]. In hypertension, gut microbial dysbiosis and its potential relationship with blood-pressure regulation have been described [14]. In preclinical models, anthocyanin-enriched purple potato flour lowered blood pressure, accompanied by enrichment of bile acid-related metabolic pathways and short-chain fatty acid-producing taxa [18]. In obesity and the attendant insulin resistance, the balance between potentially favourable and adverse microbiota-derived metabolites, including bile acids, may be altered [16], and obesity-related dysbiosis can perturb bile acid metabolism [17]. Predominantly animal studies have reported improved metabolic phenotypes following selected seaweed-derived interventions, accompanied by changes in gut microbial composition and, in some models, bile acid-related metabolism [19]. Preclinical studies further suggest that this axis is modifiable: transplantation of microbiota from healthy donors into progeroid mice restored secondary bile acid metabolism and ameliorated ageing-related phenotypes [20]. Collectively, these observations provide a rationale for investigating the axis in early cardiometabolic risk states, but they do not establish a clinically validated treatment window or support microbiome-guided primary prevention.

### Progression to overt cardiovascular events and complications

By remodelling the bile acid profile—particularly the levels of secondary bile acids such as deoxycholic acid (DCA)—gut dysbiosis has been experimentally linked to platelet activation and thrombus formation, thereby providing a plausible connection between chronic metabolic inflammation and acute cardiovascular events [21, 22]. A finely resolved regulatory axis has been delineated: *Bacteroides vulgatus* → DCA → platelet TGR5. Patients with coronary artery disease show not only reduced serum DCA but also a marked depletion of *B. vulgatus* in the gut [21]. Mechanistically, DCA engages TGR5 on the platelet surface to suppress agonist-induced platelet activation and in vivo thrombosis; genetic deletion or pharmacological blockade of TGR5 abolishes this protection entirely [21]. By linking a specific bacterial taxon, a defined bile acid species and platelet TGR5 signalling, the *Bacteroides vulgatus*–DCA–TGR5 pathway provides a mechanistic model for investigating thrombotic susceptibility in coronary artery disease [21]. It also provides a rationale for preclinical testing of approaches intended to restore this pathway; however, the efficacy and safety of probiotic supplementation, bile acid administration or faecal microbiota transplantation for cardiovascular prevention have not been established.

Additional experimental studies indicate that dietary or pharmacological manipulation can modify components of the gut microbiota–bile acid axis and attenuate atherosclerotic phenotypes. In ApoE−/− mice, capsaicin remodelled the gut microbiota, altered DCA and cholic acid levels and reduced atherosclerotic burden [23]. In another preclinical model, the EphA2 inhibitor ALW-II-41-27 altered microbial composition and secondary bile acid production, while faecal microbiota transplantation experiments supported a microbiota-dependent component of the observed effect [24]. Collectively, these findings provide preclinical mechanistic support for further investigation of the axis in atherosclerosis and atherothrombosis, but they do not establish therapeutic efficacy in humans.

From atrial fibrillation to heart failure, gut dysbiosis and disrupted bile acid signalling—exemplified by reduced fibroblast growth factor 19 (FGF19)—are increasingly implicated as potential contributors to impaired cardiac electrical stability and contractile function [25–27]. In patients with atrial fibrillation, the microbial conversion of primary to secondary bile acids is disrupted, lowering the faecal proportion of secondary bile acids and reducing circulating FGF19, a hormone induced through bile acid–FXR signalling [27]. Cellular studies show that FGF19 acts via PPARα to shield atrial cardiomyocytes from lipid accumulation, calcium-handling abnormalities, and inflammation, suggesting that suppression of this axis could contribute to atrial electrical and structural remodelling [27]. In heart failure, the fall in cardiac output produces splanchnic congestion and barrier dysfunction, which in turn worsen dysbiosis and the translocation of bacterial products; the resulting systemic inflammation further compromises cardiac performance, establishing a self-reinforcing heart–gut vicious cycle [26, 28, 29]. In patients with chronic heart failure, reduced primary bile acids and an increased secondary-to-primary bile acid ratio have been reported, with the latter associated with mortality in univariate analysis [30]. These observations document disease-associated bile acid remodelling but do not establish independent prognostic value or an upstream causal mechanism. Within this loop, microbiota-derived metabolites, including bile acids, may contribute to myocardial metabolic stress, fibrosis and inflammation; however, direct causal evidence in human heart failure remains limited [25, 26, 28]. The relevance of this dysregulation may extend to the right heart: patients with pulmonary arterial hypertension show altered gut microbial and circulating metabolite profiles, whereas intermittent fasting improved right ventricular function alongside changes in microbial metabolites, including bile acids, in a preclinical pulmonary hypertension model [31, 32]. Taken together, these observations are compatible with a contributory role for the gut microbiota–bile acid axis in arrhythmia and heart failure, but reverse causation remains plausible, particularly because congestion, altered diet, reduced physical activity and medication exposure can themselves reshape the gut environment. Within a 3PM framework, the axis should therefore be regarded as a candidate monitoring and intervention layer for secondary-prevention research rather than as an established therapeutic target.

Taken together, the available evidence suggests that gut microbiota–bile acid axis dysregulation can be detected from subclinical metabolic and cardiovascular perturbations to overt cardiovascular events. Mechanistic evidence is currently most developed for atherosclerosis and atherothrombosis, where microbial taxa, bile acid species and host receptors have been linked in relatively well-defined pathways. By contrast, evidence for atrial fibrillation, heart failure, pulmonary hypertension and cerebrovascular outcomes remains more heterogeneous and should be interpreted as emerging or hypothesis-generating. This uneven evidence landscape provides the rationale for examining the mechanistic hubs of the axis in the following section.

**Fig. 2:**
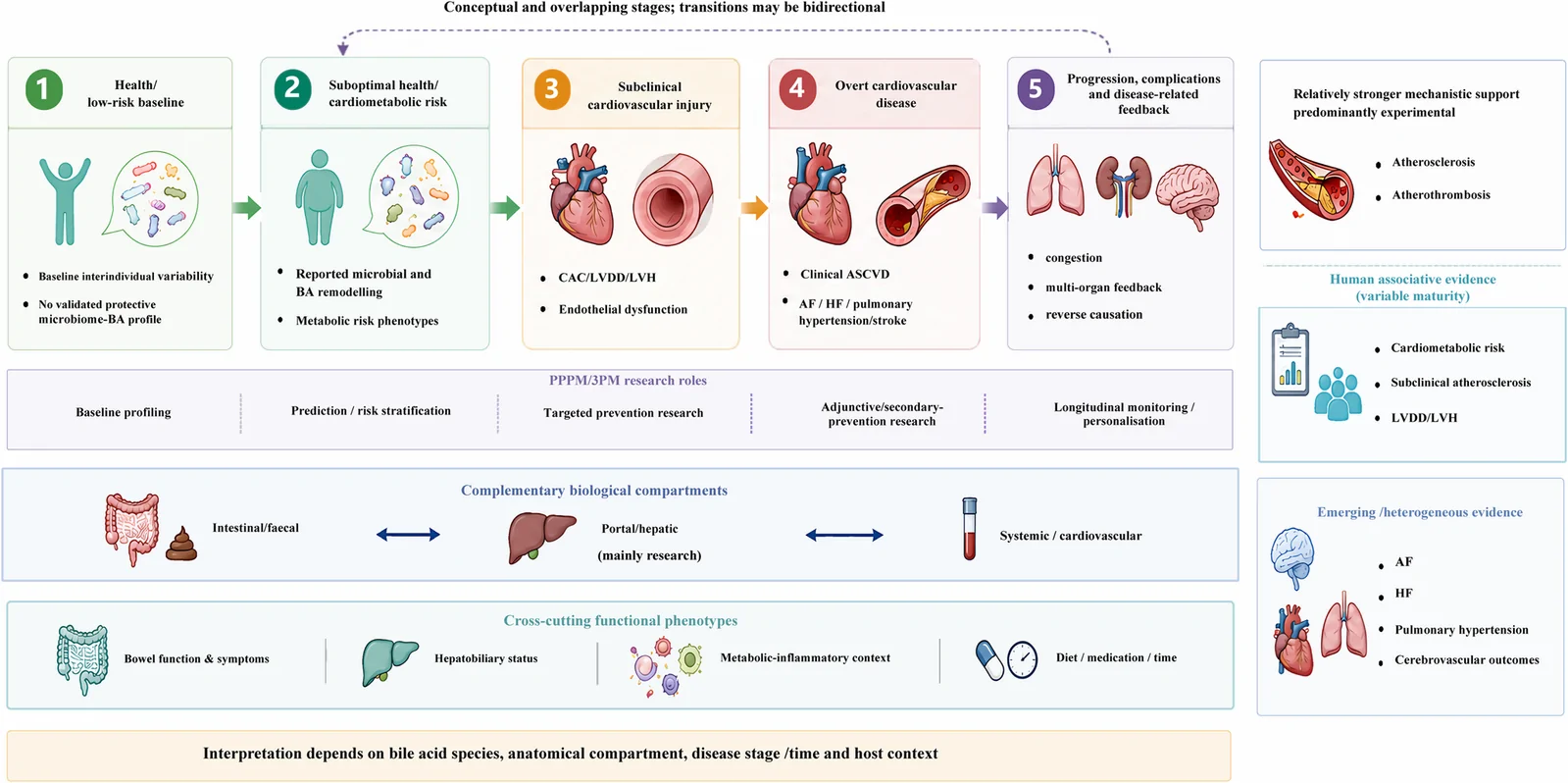
Spatiotemporal and phenotypic framework across the health–suboptimal health–cardiovascular disease continuum. The five stages are conceptual and overlapping, and transitions may be bidirectional rather than representing a validated linear natural history. The framework aligns cardiovascular stages with candidate PPPM/3PM research roles, complementary biological compartments, cross-cutting functional phenotypes and phenotype-specific evidence maturity. Mechanistic support is relatively stronger for atherosclerosis and atherothrombosis, while human evidence for cardiometabolic, subclinical and other cardiovascular phenotypes remains variable or emerging. AF, atrial fibrillation; ASCVD, atherosclerotic cardiovascular disease; CAC, coronary artery calcium; HF, heart failure; LVDD, left ventricular diastolic dysfunction; LVH, left ventricular hypertrophy; PPPM/3PM, predictive, preventive and personalised medicine

Figure 2 summarises the conceptual spatiotemporal and phenotypic framework across the health–suboptimal health–cardiovascular disease continuum, together with corresponding PPPM/3PM research roles and phenotype-specific evidence maturity. Table 1 presents an evidence-to-translation matrix aligning major cardiovascular contexts with representative evidence, microbiome–bile acid features, host phenotypes or pathways, evidence status, and PPPM/3PM interpretation and limitations.

**Table 1:** Evidence-to-translation matrix for the gut microbiota–bile acid axis across cardiovascular contexts

Cardiovascular context	Representative evidence	Microbiome–bile acid feature	Main host phenotype or pathway	Evidence status	PPPM/3PM interpretation and limitation
Suboptimal health / subclinical atherosclerosis	Human cross-sectional multi-omics association of gut *Streptococcus* spp. with coronary artery calcium in asymptomatic adults [11]	*Streptococcus* spp.; higher secondary bile acids; lower indolepropionic acid	Subclinical coronary calcification and associated metabolic profile	Moderate human associative evidence; no temporal or predictive validation	Candidate risk-marker domain for prospective validation; incremental value beyond established cardiovascular models is unknown
Early cardiac structural or functional abnormalities	Human observational associations of left ventricular diastolic dysfunction and hypertensive left ventricular hypertrophy with microbial and bile acid changes [12, 13]	Reduced *Intestinimonas massiliensis*; altered secondary bile acids; *Faecalitalea*/*Turicibacter*-related signals	Diastolic dysfunction, hypertrophic remodelling and metabolic stress	Emerging human associative evidence	Candidate contextual monitoring domain; temporality, causality and predictive performance remain unresolved
Cardiometabolic risk states	Mixed human and preclinical evidence in metabolic syndrome, obesity and hypertension [14–19]	Remodelling of bile acid pools and risk-state-associated dysbiosis	Lipid metabolism, blood-pressure regulation and systemic inflammation	Heterogeneous associative and preclinical evidence	Relevant context for prevention research; microbiome–bile acid-guided intervention selection remains investigational
Atherosclerosis	Predominantly animal mechanistic or intervention studies, including patient-derived microbiota transfer experiments [23, 24, 33–43]	BSH-mediated deconjugation; downstream bile acid transformations; FXR–FGF15/19–CYP7A1 signalling	Cholesterol catabolism, macrophage inflammation, barrier and endothelial dysfunction	Relatively stronger mechanistic evidence, predominantly preclinical	Candidate mechanistic intervention domain; human causal evidence and validated treatment-selection pathways are lacking
Atherothrombosis / coronary artery disease thrombotic risk	Human observations and experimental validation of the *Bacteroides vulgatus*–DCA–platelet TGR5 pathway [21]	Depleted *B. vulgatus*; reduced DCA; altered platelet TGR5 signalling	Platelet activation and thrombosis	Relatively stronger pathway-specific evidence; clinical translation remains unvalidated	Candidate secondary-prevention research pathway; intervention efficacy and safety have not been established
Atrial fibrillation	Human association and cardiomyocyte experiments linking suppression of the bile acid–FGF19 axis to atrial stress [27]	Reduced secondary bile acids and circulating FGF19	Cardiomyocyte lipid handling, calcium-related signalling and inflammation	Emerging human and cellular evidence	Candidate molecular stratification context; causal and predictive validation is lacking
Heart failure	Human observational evidence and mechanistic inference linking altered bile acid profiles with heart–gut feedback [26, 28–30]	Altered bile acid profiles; increased secondary-to-primary bile acid ratio; dysbiosis	Congestion, barrier dysfunction, systemic inflammation and myocardial stress	Emerging evidence with substantial reverse-causation risk	Candidate contextual monitoring domain; not an established therapeutic target
Pulmonary hypertension / right ventricular dysfunction	Human microbiome–metabolite observations and preclinical intermittent-fasting evidence [31, 32, 44]	Dysbiosis and altered circulating microbial metabolites and bile acids	Right ventricular stress, inflammation and metabolic remodelling	Emerging mixed human and preclinical evidence	Hypothesis-generating domain requiring phenotype-specific and intervention validation
Cerebrovascular and post-stroke outcomes	Human associative and preclinical evidence on post-stroke bile acid redistribution and cognitive outcomes [45–47]	Redistribution of hepatic and intestinal bile acids; lower circulating UDCA in selected post-stroke cohorts	Neuroinflammation, cognition and gut–brain/vascular feedback	Hypothesis-generating mixed human and preclinical evidence	Candidate post-stroke monitoring or adjunctive-research domain; no validated predictive or therapeutic pathway

Note. Evidence status is comparative within this Review and reflects study design, human replication, mechanistic specificity and intervention evidence. It does not imply definitive causality, validated cardiovascular risk prediction or clinical readiness. Cross-cutting gastrointestinal, hepatobiliary, molecular and exposure-related phenotypes are summarised separately in Table 2. Abbreviations: *BA* bile acid, *BSH* bile salt hydrolase, *CVD* cardiovascular disease, *DCA* deoxycholic acid, *FGF19* fibroblast growth factor 19, *FXR* farnesoid X receptor, *TGR5* Takeda G protein-coupled receptor 5, also known as GPBAR1, *UDCA* ursodeoxycholic acid

## Mechanistic hubs linking the gut microbiota–bile acid axis to cardiovascular pathology

### Receptor hubs and metabolic reprogramming

Among the principal bile acid-sensing pathways, TGR5 and FXR translate microbiota-modified bile acid signals into intracellular programmes involved in lipid and glucose metabolism and immune regulation [48–50]. TGR5, also known as G protein-coupled bile acid receptor 1 (GPBAR1), is broadly expressed across multiple tissues, including immune cells, whereas FXR is preferentially expressed in the liver and intestine [48, 49]. Dietary lipid quantity and quality can alter bile acid secretion and exert selective pressures on intestinal microbial communities, thereby modifying the substrate environment for downstream microbial bile acid transformation [51]. At the core of this axis is the canonical FXR–FGF15/19–CYP7A1 negative-feedback loop, which regulates hepatic bile acid synthesis from cholesterol [33, 34]. In this notation, FGF15 refers to the murine orthologue, whereas FGF19 denotes the human counterpart. Microbial bile salt hydrolase-mediated deconjugation generates unconjugated substrates that may undergo downstream transformations, including 7α-dehydroxylation and the formation of selected secondary bile acids, thereby modifying the bile acid signals available to intestinal and hepatic receptors [34–36].

Experimental interventions illustrate how this pathway may be modified, but their findings remain model-specific. In ApoE−/− mice, naringin-induced microbiota changes were accompanied by reduced intestinal FXR–FGF15 signalling and increased hepatic *Cyp7a1* expression, a pathway proposed to contribute to the reduced atherosclerotic phenotype observed in that model [33, 34]. *Bifidobacterium animalis* subsp. *lactis* F1-7 altered intestinal bile acid composition and FXR signalling in an atherosclerotic mouse model [35], while Zexie Yinchen Formula modified *Akkermansia* abundance, secondary bile acid profiles, intestinal FXR–FGF15 signalling and barrier-related measures [36]. In another experimental model, *Enterobacter aerogenes* ZDY01 reduced caecal CDCA and attenuated intestinal FXR–FGF15 signalling, with consequent upregulation of *Cyp7a1* [37]. This strain should be interpreted as a mechanistic proof-of-concept rather than as a clinically applicable probiotic. *Aloe vera* polysaccharide improved lipid-related and atherosclerotic phenotypes in ApoE−/− mice alongside changes in hepatic FXR-related signalling and bile acid transport [38]. Polyphenols have also been reviewed as candidate modifiers of CYP7A1 and bile acid metabolism through FXR, NF-κB and related pathways [39]. Taken together, FXR and TGR5 should be viewed as major bile acid-sensing pathways whose effects depend on the intervention, anatomical compartment and experimental context.

Disruption of microbiota-related metabolic signalling may impair cellular lipid handling and stress adaptation, although the available evidence is pathway- and model-specific. In patients with atrial fibrillation, reduced secondary bile acid production was accompanied by lower circulating FGF19; in vitro, FGF19 acting through PPARα reduced palmitate-induced lipid accumulation and abnormal Ca²⁺/calmodulin-dependent protein kinase II phosphorylation in atrial cardiomyocytes [27]. These findings support a link between the bile acid–FXR–FGF19 axis and cardiomyocyte lipotoxic stress, but they do not establish generalised endoplasmic-reticulum stress, mitochondrial dysfunction or autophagic failure in the human myocardium. In ApoE−/− mice, myricetin remodelled the gut microbiota and increased hepatic CYP7A1 and CYP8B1 expression [40], supporting modulation of cholesterol–bile acid metabolism rather than direct rescue of organelle injury.

From a 3PM perspective, mitochondrial functional read-outs could be explored as complementary research measures of subclinical energetic stress and intervention response [52, 53]. Their integration with microbiome–bile acid signatures remains hypothetical and requires direct validation in longitudinal cardiovascular studies.

### Context-dependent and compartment-specific effects of bile acids

A critical issue in interpreting the gut microbiota–bile acid axis is that bile acids should not be viewed as uniformly protective or uniformly harmful metabolites. Their biological effects depend on molecular species, concentration, tissue compartment, receptor distribution, disease stage, and the surrounding inflammatory or metabolic milieu. For example, DCA may suppress platelet activation through platelet TGR5 signalling in the context of atherothrombosis, whereas experimental administration of DCA has also been associated with increased myocardial contractility, cardiac output, and arterial pressure [21, 54]. These observations are not necessarily contradictory; rather, they suggest that the same bile acid may exert divergent effects depending on whether the dominant target is a platelet receptor, vascular or myocardial tissue, hepatic metabolism, or intestinal signalling.

This context dependency is particularly important for FXR- and TGR5-mediated signalling. Intestinal FXR inhibition may enhance hepatic cholesterol-to-bile acid conversion by releasing feedback suppression of CYP7A1, whereas hepatic FXR activation may produce distinct metabolic consequences [49, 50]. Similarly, selected secondary bile acids may support barrier or anti-inflammatory signalling under specific physiological conditions, whereas excessive or compartmentally inappropriate exposure may contribute to inflammatory activation, barrier disruption or vascular dysfunction [49, 55]. Accordingly, future studies should pre-specify the bile acid species and sampled compartment, the intended receptor or metabolic pathway, and the hypothesised direction of change, rather than assume that increasing or decreasing total or secondary bile acids will be beneficial. Whether this function-informed approach improves biological or clinical outcomes remains unproven. Where feasible, studies should distinguish faecal, intestinal, portal, systemic, hepatic and cardiovascular tissue bile acid pools and relate each pool to receptor activation, immune tone, endothelial function, platelet activity and cardiovascular phenotype.

The biological consequences of bile acid signalling extend beyond cardiovascular and metabolic tissues. Experimental and clinical observations link bile acid composition and enterohepatic feedback to intestinal secretion, gastrointestinal transit, epithelial barrier function and sensory signalling [56–59]. These observations indicate that gastrointestinal and gut–brain functional phenotypes may provide important interpretive context for microbiome–bile acid profiles in cardiovascular cohorts.

### Immune–barrier–endothelial responses

Microbiota-modified bile acids can modulate immune-cell activation, differentiation and cytokine production through receptor-dependent pathways [55, 60]. In a murine liver ischaemia–reperfusion model, antibiotic pretreatment altered the hepatic bile acid pool, including unconjugated bile acids such as ursodeoxycholic acid, and activated FXR. Activated FXR subsequently bound an FXR-response element within the TLR4 promoter and suppressed downstream MAPK and NF-κB signalling [61]. Because this evidence derives from a non-cardiovascular hepatic injury model, its relevance to cardiovascular inflammation remains indirect. Under dysbiotic conditions, altered bile acid profiles and other microbial metabolites may modulate innate immune pathways and sustain low-grade inflammation relevant to atherogenesis [55].

Dietary patterns may modify this inflammatory context. Mediterranean-style diets have been associated with changes in gut microbial composition, microbial metabolites and inflammatory pathways, including NLRP3- and TLR4-related signalling [62]. However, the extent to which these changes mediate cardiovascular benefit through the microbiota–bile acid axis remains uncertain. A systematic review of prospective cohorts reported associations between several microbiota-related metabolites and cardiovascular outcomes; however, findings for secondary bile acids were inconsistent. These observations support the epidemiological relevance of microbial metabolites without establishing causality or a central pathogenic role [63].

Physiological bile acid signalling and microbiota-derived metabolites may support intestinal barrier homeostasis and endothelial function, whereas disruption of these systems has been associated with increased intestinal permeability and vascular dysfunction [41, 44, 49, 64]. Bile acid signalling contributes to barrier regulation, but epithelial integrity is also shaped by microbial, immune, dietary and host factors and should not be attributed to bile acids alone. In a preclinical model, chronic apical periodontitis altered gut microbial composition and bile acid metabolism, impaired mucus and tight-junction markers, and aggravated atherosclerotic lesions, providing experimental support for an oral–gut–vascular interface [64]. Once barrier function is impaired, translocation of bacterial products may promote systemic inflammation and reduce nitric oxide bioavailability. Preclinical intervention studies provide mechanistic support for this pathway: inulin-type fructans improved endothelial dysfunction in ApoE−/− mice in association with microbiota remodelling, altered bile acid turnover and nitric oxide synthase-dependent effects [41]. Ginsenoside- and astaxanthin-based interventions have also improved barrier-related and atherosclerotic phenotypes in experimental models [42, 65], but their relevance to human cardiovascular prevention remains unconfirmed. This dysregulation is by no means confined to atherosclerosis: patients with pulmonary arterial hypertension also display a pro-inflammatory dysbiotic microbiota together with relatively low circulating secondary bile acids, suggesting that microbiota–bile acid axis dysfunction may be a shared feature of the pathological networks underlying diverse cardiovascular diseases [44].

## Systemic crosstalk, functional phenotypes and environmental interfaces

### Gut–liver–cardiovascular metabolic crosstalk

Bacterial bile salt hydrolases catalyse the deconjugation of conjugated bile acids, generating unconjugated substrates that can undergo downstream microbial transformations, including 7α-dehydroxylation and the formation of selected secondary bile acids [66, 67]. Differences in BSH abundance, catalytic activity and phylogenetic distribution can therefore reshape intestinal bile acid composition and modify intestinal FXR–FGF15/19 signalling, with downstream effects on hepatic CYP7A1-mediated bile acid synthesis and cholesterol homeostasis [34, 42, 66, 67]. Loss-of-function evidence further supports the importance of this circuit: antibiotic-induced depletion of the gut microbiota disrupts enterohepatic bile acid circulation and worsens hypercholesterolaemia, an effect that resolves after microbiota recovery [68]. Fibre-rich dietary components and bile acid sequestrants may modify faecal bile acid loss, intestinal cholesterol handling and hepatic cholesterol disposal, whereas microbiome-targeted approaches remain investigational [69, 70].

Beyond long-term metabolic regulation, selected bile acids can exert acute vascular or myocardial effects, while the gut microbiota may influence autonomic tone through the gut–brain axis [14, 54, 71]. Intravenous deoxycholic acid (DCA) increased myocardial contractility, cardiac output and arterial pressure in rats, demonstrating that exogenous DCA can acutely alter haemodynamics [54]. Whether endogenous microbiota-derived DCA has comparable physiological or clinical effects remains unknown. In a separate preclinical example, stachyose improved obesity-related phenotypes and promoted white adipose tissue browning in high-fat diet-fed mice alongside changes in gut microbial composition [72], although bile acid mediation was not established.

Gut chemosensory pathways add another regulatory layer. Loss of the SCFA receptor GPR41 alters gut microbial composition, bile acid profiles and intestinal NPC1L1 expression, thereby reducing plasma LDL cholesterol in mice [73]. Although this pathway operates partly outside the classical BSH–FXR axis, it highlights how microbial metabolite sensing within the intestine may influence bile acid metabolism, cholesterol absorption and interindividual variability in lipid responses.

### Bile acid-related gastrointestinal and gut–brain functional phenotypes

Although the present Review remains centred on cardiovascular disease, microbiota–bile acid dysregulation may also be expressed clinically through gastrointestinal and gut–brain functional phenotypes. Within the intestine, bile acid species and luminal exposure influence secretion, epithelial integrity, gastrointestinal transit and sensory signalling. In experimental models, TGR5/GPBAR1 activation promotes colonic motility, whereas TGR5 deficiency delays intestinal transit [56]. Intestinal FXR–FGF19 feedback, in parallel, links ileal bile acid sensing to hepatic bile acid synthesis [49, 50]. These effects further illustrate why total or secondary bile acid concentrations cannot be interpreted independently of molecular species, anatomical compartment and host context.

Disorders of gut–brain interaction provide a clinically recognisable example of this heterogeneity. In a large cohort of patients with diarrhoea-predominant irritable bowel syndrome, approximately one quarter had excessive faecal bile acid excretion accompanied by higher serum 7α-hydroxy-4-cholesten-3-one (C4), lower FGF19 and enrichment of bile acid-transforming Clostridia [57]. Conversely, reduced faecal bile acid excretion has been identified in a subgroup of patients with constipation-predominant IBS [58]. A smaller human study combined with microbiota-humanised rat experiments further linked alterations in the microbiota–bile acid–TGR5 axis to barrier impairment and visceral hypersensitivity in IBS-D [59]. These findings do not support treating IBS as a single bile acid-defined disorder; instead, they illustrate the need to stratify patients according to bowel phenotype and bile acid function.

From a PPPM perspective, these gastrointestinal and gut–brain features should be incorporated as cross-cutting contextual variables rather than as additional cardiovascular endpoints. Clinically accessible assessment may include stool form and frequency, abdominal pain or bloating, relevant dietary exposure, and medications that alter bowel function or enterohepatic circulation. In specialist or research settings, this may be extended to serum C4 and FGF19, targeted faecal or plasma bile acid profiling, intestinal transit assessment, and metagenomic estimates of bile acid-transforming capacity [57–59, 66, 67]. Candidate research panels could additionally integrate circulating microbial metabolites, systemic inflammatory markers, standardised intestinal permeability assessments, and autonomic or neuroimmune read-outs. However, these measures are biologically non-specific, may be influenced by diet, medication exposure, comorbidities and disease severity, and currently lack harmonised analytical methods and clinically meaningful thresholds. None is validated as a specific marker of bile acid-mediated gut–brain signalling or for cardiovascular risk stratification.

In cardiovascular cohorts, these data may help determine whether an observed microbiome–bile acid signature reflects altered intestinal transit, hepatobiliary comorbidity, diet or medication exposure, primary alterations in microbial metabolism, or secondary effects of established CVD. However, current evidence does not establish that IBS phenotypes independently predict cardiovascular events or that correcting bile acid-related bowel dysfunction improves cardiovascular outcomes. Gastrointestinal and gut–brain phenotypes should therefore be regarded as candidate contextual, stratification and monitoring variables requiring prospective validation rather than as established cardiovascular biomarkers.

### Environmental and multi-organ interfaces

The gut microbiota–bile acid axis may also integrate inflammatory and environmental inputs beyond diet. Oral inflammation provides one example of such cross-system communication: chronic oral infection or colonisation by oral pathogens can promote gut dysbiosis, disturb bile acid metabolism, impair intestinal barrier function and aggravate atherosclerotic lesions, supporting the concept of an oral–gut–vascular axis [64, 74]. This evidence suggests that oral health may contribute to systemic microbial ecology and cardiovascular risk, although its clinical relevance for axis-guided prevention still requires validation.

Non-dietary stressors further illustrate the plasticity of the axis within a 3PM framework. Sleep disturbance, intermittent hypoxia, ageing and post-stroke states have each been associated with shifts in gut microbial taxa and bile acid profiles, suggesting that the axis may act as an interface through which behavioural, environmental and disease-related stressors influence cardiometabolic risk [45, 75–77]. For example, chronic insomnia has been linked to cardiometabolic disease risk partly through microbial and secondary bile acid changes, while intermittent hypoxia models show microbiota-dependent acceleration of atherosclerosis accompanied by altered bile acid profiles [75, 76]. Acute cardiovascular events may also reshape the axis, as post-stroke models show concomitant dysbiosis and redistribution of hepatic and intestinal bile acids [45]. A recent translational study also associated lower circulating ursodeoxycholic acid with post-stroke cognitive impairment in patients and reported improved cognitive outcomes after ursodeoxycholic acid administration in stroke mice [46]. These findings are hypothesis-generating and do not establish clinical efficacy.

Translational evidence supports the relevance of this systemic interface: transfer of gut microbiota from patients with coronary artery disease into mice increases circulating cholesterol and induces vascular dysfunction through remodelling of the secondary bile acid pool and suppression of hepatic bile acid synthesis [43]. More broadly, environmental and behavioural factors associated with urbanisation, including pollutants and lifestyle transitions, may perturb microbial metabolites such as bile acids and thereby influence cardiometabolic health [78]. These observations should be regarded as emerging evidence rather than mature clinical targets, but they broaden the 3PM view of the gut microbiota–bile acid axis from diet-centred modulation to multi-exposure, multi-organ risk management.

## Targeted prevention and responder-stratified personalisation of the gut microbiota–bile acid axis

Within the PPPM/3PM framework, modulation of the gut microbiota–bile acid axis should not be viewed as a uniform therapeutic goal. Future studies should consider cardiovascular stage, baseline molecular and functional phenotypes, and a prespecified preventive or therapeutic objective. In early-risk states, established healthy dietary measures provide the clinically accessible prevention context, whereas microbiome- or bile acid-guided nutrition and microbial interventions remain investigational. In established cardiovascular disease, microbiota- and bile acid-directed approaches should be evaluated as adjunctive research strategies alongside guideline-directed care. Longitudinal assessment and prespecified responder analyses should be incorporated into future intervention studies to evaluate biological benefit, non-response and potentially maladaptive changes.

### Diet, precision nutrition and candidate response stratification

Established healthy dietary patterns, particularly Mediterranean-style patterns and improvements in overall diet quality, are the most clinically mature preventive approaches discussed in this Review [79]. These dietary patterns may modify gut microbial and metabolite profiles [62, 79–82], but the extent to which their cardiovascular benefits are mediated specifically through bile acid remodelling remains uncertain. Intermittent fasting should be considered separately because much of the evidence linking it to microbiota–bile acid changes and cardiovascular phenotypes derives from preclinical models [32, 83]. Within a PPPM/3PM framework, diet therefore provides a clinically accessible prevention context in which candidate microbiome–bile acid response modifiers can be prospectively evaluated, rather than a validated basis for phenotype-guided dietary prescription.

Clinical and translational studies indicate that dietary interventions can modify components of the gut microbiota–bile acid axis, but they do not yet establish axis-guided treatment selection. In the DIRECT-PLUS trial, baseline faecal bile acid signatures were associated with heterogeneity in weight-loss response, while on-treatment bile acid changes correlated with changes in body mass index and lipid profile [84]. These observations support prospective evaluation of bile acid profiles as candidate response modifiers but do not establish clinically usable treatment-selection markers. A caregiver–child dietary intervention further showed that improved diet quality was accompanied by increased faecal excretion of microbially produced lithocholic acid and reductions in blood pressure, supporting the broader relevance of diet–microbiota–bile acid interactions for cardiometabolic research [85]. Time-restricted dietary interventions may also reshape the gut microbiota and alter bile acid profiles, including tauroursodeoxycholic acid levels, although much of the axis-specific evidence remains preclinical [32, 83].

These observations motivate research into precision nutrition, but they do not yet justify microbiome- or bile acid-guided dietary assignment. Interindividual variability in baseline microbial composition, bile acid-transforming capacity and faecal bile acid profiles may contribute to heterogeneity in dietary response [84, 86]. Future trials should test whether these molecular features improve intervention selection or prediction of biological response beyond conventional clinical and dietary information. Until such evidence is available, microbiome and bile acid profiling should be regarded as investigational rather than used routinely to assign Mediterranean-style diets, fibre or prebiotic supplementation, intermittent fasting or other nutrition-based interventions. This question may be particularly relevant in metabolic dysfunction-associated steatotic liver disease, in which diet, gut microbiota, hepatobiliary status and bile acid metabolism are closely interconnected [87].

Precision nutrition should distinguish dietary modifiers with direct relevance to bile acid biology from exposures supported mainly by microbiome, inflammatory or epidemiological evidence. Fibre type and dose can influence microbial fermentation, bile acid deconjugation and faecal excretion, whereas polyphenol-rich foods may modify microbial ecology and bile acid-related signalling [39, 69, 88–90]. Dietary fat quality influences bile acid secretion and the selective pressure exerted on the intestinal microbiota [51]. Feeding–fasting timing should also be recorded as a potential modifier of microbiome and bile acid dynamics. Human experimental data demonstrate robust 24-hour rhythms in circulating bile acids and show that these temporal relationships are sensitive to environmental timing cues and sleep disruption [91]; however, cardiovascular trials of bile acid-guided meal timing remain lacking.

Evidence for fermented foods and coffee is less directly linked to the cardiovascular microbiota–bile acid axis. In a prospective human dietary intervention, a fermented-food-rich diet increased microbiome diversity and altered several inflammatory measures, but the study did not establish bile acid mediation or cardiovascular benefit [92]. Coffee consumption has been associated reproducibly with specific microbial features across multiple cohorts, including enrichment of *Lawsonibacter asaccharolyticus* [93]; however, its effects on bile acid pools and its utility as an axis-guided cardiovascular intervention remain uncertain. Fermented foods and coffee should therefore initially be treated as measured exposures and candidate response modifiers rather than as universally recommended microbiome therapies.

Future precision-nutrition trials should pre-specify the dietary component and dose, baseline microbiome–bile acid and clinical phenotype, adherence measure, mechanistic endpoint and cardiovascular or cardiometabolic outcome. Existing evidence supports established healthy dietary patterns more strongly than microbiome-guided dietary prescription. Baseline microbial and bile acid signatures should therefore be prospectively evaluated as candidate stratification and response-monitoring variables rather than assumed to be clinically actionable.

### Microbial, bile acid-directed and pharmacological strategies

Natural products, phytochemicals and traditional herbal formulas provide one group of mechanistic examples for multi-node regulation of the gut microbiota–bile acid axis. Polyphenols, flavanones, ginsenosides and selected herbal formulas have been reported to reshape microbial composition, modify bile acid pools and regulate FXR–FGF15/19–CYP7A1 signalling in experimental models [39, 90, 94–99]. These findings are useful for identifying candidate regulatory nodes; however, their translation into cardiovascular PPPM requires standardised formulations, dose–response evaluation, safety assessment, human pharmacokinetic characterisation and cardiovascular endpoint validation. Therefore, they should be presented as hypothesis-generating or adjunctive strategies rather than as established clinical interventions.

Representative examples illustrate this multi-node regulation. Naringin appears to act largely within the gut, where it remodels the microbiota and modulates the FXR–FGF15–CYP7A1 axis, whereas naringenin may exert more direct hepatic effects after absorption [33, 34]. Ginsenosides may combine microbiota-mediated bile salt hydrolase regulation with barrier-supportive effects involving *Akkermansia muciniphila* [42]. Selected traditional formulas, including Zexie Yinchen Formula and Simiao Pill, have also been reported to modulate *Akkermansia* abundance, bile acid composition and FXR–FGF15/19–CYP7A1 signalling in experimental models [36, 97]. Specific bile acid analogues, such as glycoursodeoxycholic acid, and dietary restriction paradigms, such as methionine restriction, provide additional preclinical examples of bile acid-linked microbial modulation in atherosclerosis models [100, 101]. Nevertheless, these interventions should be considered candidate strategies that require human validation rather than ready-to-use cardiovascular therapies.

Microbiota-directed interventions more directly target upstream functions of the axis, but taxonomic identity should be regarded as a necessary descriptor rather than a sufficient selection criterion. Candidate strains should be characterised at strain level for bile salt hydrolase isoenzyme and substrate specificity; bile acid-inducible pathway activity, 7α-dehydroxylation and other bile acid-transforming functions; the direction and anatomical compartment of the resulting bile acid changes; effects on FXR/TGR5 signalling, epithelial barrier integrity and inflammatory responses; ecological persistence; and antimicrobial-resistance and safety profiles [66, 67, 102–104]. Prebiotic selection should similarly be linked to the substrate-utilisation capacity of the baseline microbial community and to the specific metabolic function intended to be modified. Importantly, bile salt hydrolase activity should not be classified as inherently beneficial, because its consequences depend on substrate preference, downstream microbial conversion, intestinal transit and host enterohepatic feedback.

Within this function-informed framework, *Bifidobacterium animalis* subsp. *lactis* F1-7 and *Bacteroides vulgatus* are best regarded as mechanistic exemplars rather than treatment recommendations. The former altered intestinal bile acid–FXR signalling in an atherosclerotic mouse model, whereas the latter modulated DCA–platelet TGR5 signalling in experimental atherothrombosis [21, 35]. These examples demonstrate how a defined microbial function can be linked to a prespecified host pathway, but neither establishes routine probiotic or live-biotherapeutic treatment for CVD. Defined probiotics, next-generation live biotherapeutics, engineered organisms and faecal microbiota transplantation therefore remain investigational and require phenotype-stratified trials with prespecified functional targets, measures of engraftment or functional response, safety assessment and cardiovascular endpoints [102, 105, 106].

Bile acid-directed and pharmacological strategies target downstream nodes of the axis. Bile acid analogues, FXR/TGR5 modulators, enzyme inhibitors and bile acid sequestrants may influence bile acid pool composition, enterohepatic circulation, cholesterol disposal and receptor-mediated inflammatory or vascular responses [50, 70, 107–109]. Established cardiovascular drugs may also interact with this axis; for example, statins have been shown in experimental studies to alter gut microbial composition and bile acid pools through a pregnane X receptor-dependent mechanism [110]. Small molecules such as ALW-II-41-27 further suggest that pharmacological effects on atherosclerosis may be partly mediated through gut microbiota remodelling and secondary bile acid production [24]. Microbially derived metabolites, including bile acids and SCFAs, can reach the systemic circulation and affect extra-intestinal immune and cardiovascular pathways, underscoring the need for cross-organ safety and efficacy monitoring when the axis is therapeutically manipulated [2, 60, 109]. These observations broaden the candidate intervention landscape but reinforce the need for caution. Future studies should pre-specify the bile acid species and sampled compartment, the intended receptor or metabolic pathway, and the hypothesised direction of change, rather than assume that increasing or decreasing total or secondary bile acids will be beneficial. They should then test whether this function-informed approach improves biological or clinical outcomes.

Taken together, microbial, bile acid-directed and pharmacological approaches represent candidate intervention classes for secondary-prevention and adjunctive-treatment research. At present, most remain preclinical, strain-specific or insufficiently validated in axis-guided clinical trials. Future studies should prospectively test whether baseline microbiome–bile acid and functional phenotypes improve intervention selection, while prespecifying the mechanistic target, safety assessment and clinically meaningful cardiovascular outcomes.

### Digital monitoring and responder-stratified personalisation

Evaluating the potential predictive contribution of the gut microbiota–bile acid axis requires repeated observation rather than isolated stool or blood sampling. Microbial composition and bile acid pools vary with diet, sleep, physical activity, medication exposure, circadian rhythm and disease status; a single measurement may therefore fail to capture within-person variation or the biological response to intervention.

Digital tools may provide relevant temporal and exposure context. Wearable devices, home blood pressure monitoring and app-based dietary or lifestyle records can capture activity, sleep, heart-rate variability, blood pressure, dietary adherence, feeding–fasting timing, fibre and fermented-food intake, coffee exposure, bowel habit and gastrointestinal symptom trajectories [111]. These variables may aid interpretation because circulating bile acids exhibit temporal variation, while fermented-food and coffee exposures have been associated with changes in gut microbial or inflammatory profiles [91–93]. Liver biochemistry or hepatobiliary imaging should be obtained or repeated only when clinically indicated, rather than treated as high-frequency digital measures.

When longitudinal contextual data are integrated with repeated stool metagenomics, plasma or faecal bile acid profiling and prespecified inflammatory or functional measures, they may provide the basis for a candidate monitoring framework. Future studies should evaluate whether this framework detects meaningful within-person biological change, documents exposure and adherence, and identifies a prespecified molecular or physiological response.

**Fig. 3:**
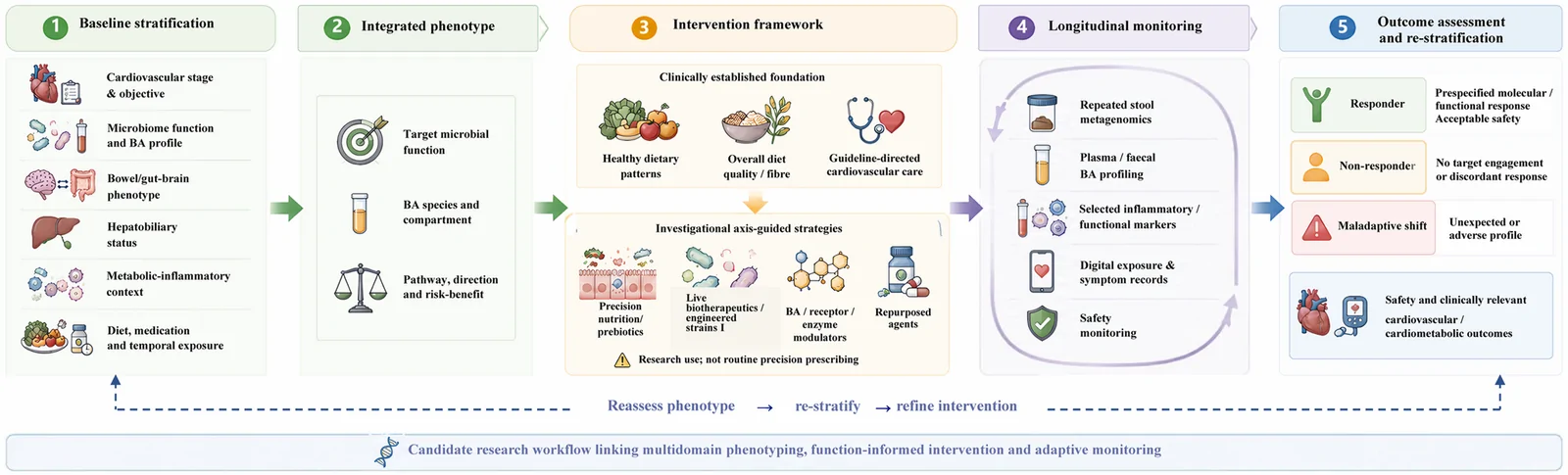
Function-informed intervention and responder-stratified monitoring framework. Multidomain baseline phenotyping informs the selection of microbial functions, bile acid species, anatomical compartments and candidate pathways for prospective evaluation. Healthy dietary patterns and guideline-directed cardiovascular care form the established clinical foundation, whereas microbiome-guided nutrition, live biotherapeutics, faecal microbiota transplantation and bile acid-directed strategies remain investigational. Longitudinal molecular, digital, functional and safety monitoring supports classification of responders, non-responders and maladaptive changes, followed by phenotype reassessment and intervention refinement. This figure represents a candidate research workflow rather than a validated treatment-selection algorithm. BA, bile acid; FMT, faecal microbiota transplantation

Future intervention studies should test whether baseline microbiome–bile acid profiles combined with multidomain clinical phenotypes improve responder classification. Follow-up profiling should classify participants as biological responders or non-responders according to prespecified molecular and clinical response criteria and should identify potentially maladaptive changes. Adaptive, responder-stratified and n-of-1 designs are research strategies for evaluating this approach rather than established routes for routine cardiovascular care. Figure 3 summarises the candidate research workflow linking multidomain baseline stratification, function-informed intervention, longitudinal monitoring, outcome assessment and responder-based re-stratification.

## Current limitations and unresolved questions

Despite growing evidence supporting the gut microbiota–bile acid axis as an integrative framework for cardiovascular disease, several limitations must be addressed before this axis can be translated into clinically actionable PPPM. These limitations should be interpreted not simply as weaknesses of the current literature, but as implementation gaps that define the next steps for predictive diagnostics, targeted prevention and personalised intervention.

First, the strength of evidence remains uneven across cardiovascular phenotypes. Mechanistic support is relatively strong for atherosclerosis and atherothrombosis, where microbial taxa, bile acid species and host receptors have been linked in relatively well-defined pathways. By contrast, evidence for atrial fibrillation, heart failure, pulmonary hypertension and cerebrovascular outcomes remains more heterogeneous and often relies on observational associations, preclinical models or indirect mechanistic inference [47, 112–115]. This uneven evidence landscape requires phenotype-specific interpretation rather than uniform extrapolation across all cardiovascular diseases.

Second, causal inference in humans remains unresolved. Dysbiosis and bile acid remodelling may act as upstream contributors to cardiovascular pathology, but they may also represent downstream consequences of altered diet, medication exposure, systemic inflammation, intestinal hypoperfusion, congestion or established disease. Cross-sectional microbiome and metabolomics studies are particularly vulnerable to reverse causation and residual confounding. Longitudinal cohorts with repeated sampling before and after disease onset are therefore needed to clarify whether specific microbial or bile acid signatures precede, accompany or follow cardiovascular progression.

Third, bile acid signalling is highly species-, compartment-, receptor- and disease-stage dependent. The same bile acid species may exert divergent effects depending on whether the dominant target is the intestine, liver, circulation, vascular wall, platelet compartment or myocardium. Therapeutic modulation should therefore not be framed as a simple increase or decrease in total or secondary bile acids. Whether strategies defined by a prespecified bile acid species, sampled compartment, intended receptor or metabolic pathway, and hypothesised direction of change improve biological or clinical outcomes remains unproven and requires prospective evaluation.

Fourth, methodological heterogeneity limits comparability and reproducibility. Current studies differ substantially in sample type, timing of collection, dietary control, sequencing platform, metabolomics method, bile acid panel coverage, normalisation strategy and bioinformatic pipeline. In addition, faecal, plasma, portal, hepatic and tissue bile acid pools may provide different biological information, but they are rarely profiled in parallel. Standardised protocols for microbiome analysis and multi-compartment bile acid profiling are needed before candidate signatures can be compared reproducibly and evaluated for predictive validity.

Fifth, clinical translation is further complicated by substantial interindividual variability and confounding. Baseline diet, geography, host genetics, age, sex, comorbidities, microbial ecology and medication exposure can all influence gut microbiota composition and bile acid metabolism. Commonly used cardiovascular and metabolic drugs, antibiotics, bile acid sequestrants and other medications may modify the axis and should be explicitly recorded and adjusted for in future studies. Without careful control of these factors, microbiota–bile acid signatures may be difficult to interpret or reproduce across populations.

Sixth, the proposed functional phenotyping layer also has important limitations. Gastrointestinal symptoms are non-specific, IBS is mechanistically heterogeneous, and serum C4, FGF19 and faecal bile acid assays are neither universally available nor fully standardised [57–59]. Putative intestinal permeability, neuroimmune and autonomic biomarkers lack validated clinical thresholds and may be influenced by multiple non-gastrointestinal factors. Conversely, liver biochemistry and imaging identify organ-level abnormalities and potential confounders but do not directly resolve bile acid pool composition, microbial transformation or receptor signalling [116, 117]. Dietary exposures such as fermented foods, coffee and feeding–fasting timing are also susceptible to measurement error and residual confounding [91–93]. These domains should therefore be evaluated as candidate contextual or stratification variables rather than presented as established cardiovascular biomarkers or population-wide screening tests.

Seventh, the present Review itself has methodological limitations. The evidence was synthesised narratively rather than through a preregistered systematic review with a formal risk-of-bias or certainty-of-evidence assessment. The literature included may therefore not be exhaustive, and the selection and weighting of evidence involved author judgement. The comparative evidence-status categories presented in Table 1 are interpretive and should not be regarded as equivalent to a formal GRADE assessment. Although the Review sought to distinguish human associative evidence from experimental mechanistic evidence, heterogeneity in terminology, assays and reporting may have influenced the synthesis. Future phenotype-specific systematic reviews should apply predefined eligibility criteria and formal evidence-appraisal methods.

Finally, axis-guided intervention studies remain insufficient. Many dietary, microbial, bile acid-directed and pharmacological interventions have been shown to modify components of the axis, but few studies have tested whether baseline microbiome–bile acid signatures can guide intervention selection or improve cardiovascular outcomes beyond conventional risk stratification. Future work should therefore move from association-based biomarker discovery to temporally resolved and intervention-linked validation. Longitudinal cohorts with repeated sampling and controlled intervention studies will be needed to establish temporality, causal relevance and biological response. Human-relevant experimental systems may support mechanistic validation, whereas prediction models should be developed, reported and evaluated according to current methodological standards to determine whether axis-informed signatures add value beyond established cardiovascular assessment [118–121].

## Conclusions and expert recommendations in the PPPM/3PM framework

The gut microbiota–bile acid axis provides a biologically plausible and clinically relevant framework for linking diet, environmental exposures and microbial metabolic reprogramming to cardiovascular health and disease. Across the cardiovascular continuum, this axis connects microbial ecology, bile acid signalling, host lipid metabolism, immune regulation, barrier integrity, platelet activity and myocardial stress [2, 109, 112]. Its relevance is most strongly supported in atherosclerosis and atherothrombosis, whereas evidence for heart failure, atrial fibrillation, pulmonary vascular disease and cerebrovascular outcomes remains more heterogeneous and should be interpreted with greater caution [47, 113–115]. Building on this synthesis, we propose the following expert recommendations organised around the three pillars of predictive, preventive and personalised medicine.

### Predictive diagnostics, functional phenotyping and risk stratification

The potential predictive contribution of the gut microbiota–bile acid axis lies in candidate molecular and functional signatures that may become detectable before overt cardiovascular disease and may support longitudinal risk research. Candidate read-outs include compartment-specific bile acid species and ratios, bile salt hydrolase and other bile acid-transforming functions, selected microbial features, and complementary endothelial or mitochondrial functional measures. These candidates should be evaluated prospectively for temporal stability, reproducibility, incremental value beyond established cardiovascular risk models and association with clinically meaningful outcomes; they should not yet be regarded as validated markers of the health-to-disease transition during suboptimal health [9]. We recommend that future longitudinal cohorts incorporate paired faecal metagenomics and plasma/faecal bile acid profiling in individuals with suboptimal health, metabolic syndrome, hypertension, subclinical atherosclerosis or other high-risk states. Candidate signatures should be evaluated against prespecified intermediate phenotypes, such as coronary artery calcium progression, endothelial function, lipid trajectories and inflammatory measures, and against clinical outcomes including incident cardiovascular events.

To operationalise this framework, a tiered phenotyping strategy is more appropriate than immediate routine multi-omics screening. Tier 1 comprises clinically accessible context: established cardiovascular and metabolic risk; bowel habit and gastrointestinal symptom patterns; dietary and medication exposures; anthropometric and metabolic measures; liver biochemistry, distinguishing hepatocellular markers such as alanine aminotransferase and aspartate aminotransferase from cholestatic markers including alkaline phosphatase, gamma-glutamyl transferase and bilirubin; and ultrasonographic assessment of hepatic steatosis, gallbladder disease or biliary dilatation when clinically indicated [116, 117]. Elastography may be added when the probability of hepatic fibrosis warrants further assessment. These measurements may identify hepatobiliary comorbidity and major confounders of bile acid profiles, but neither routine liver biochemistry nor ultrasound directly measures bile acid synthesis, microbial transformation or receptor activity.

Tier 2, for selected cohorts or specialist evaluation, may comprise serum C4 and FGF19, targeted plasma and faecal bile acid panels, and metagenomic or metatranscriptomic measures of bile acid-transforming functions [57, 58, 66, 67]. The established clinical relevance of C4 and FGF19 is primarily related to bile acid synthesis and bile acid-related gastrointestinal phenotypes; their value for cardiovascular risk stratification remains unvalidated. Tier 3 remains research-oriented and may include intestinal transit or permeability testing, neuroimmune or autonomic read-outs, multi-omics integration and longitudinal digital measures. No composite microbiome–bile acid–clinical phenotype panel is currently validated to improve cardiovascular risk prediction beyond established clinical models; its incremental value, analytical reproducibility, feasibility and cost-effectiveness require prospective evaluation. The proposed multidomain phenotyping domains, their intended interpretive roles and principal limitations are summarised in Table 2.

**Table 2:** Proposed multidomain functional phenotyping framework for prospective evaluation of the gut microbiota–bile acid axis in cardiovascular PPPM

Domain	Tier 1: clinically accessible context and standardised sampling	Tier 2/3: specialist or research measures	Proposed interpretive role and principal limitation
Cardiovascular–metabolic and inflammatory context	CVD stage; blood pressure; lipids; glucose/HbA1c; adiposity; comorbidities; clinically indicated routine inflammatory measures or prespecified research biomarkers	Endothelial or vascular functional testing; expanded metabolic and inflammatory profiling	Anchors microbiome–bile acid data to established cardiovascular risk and systemic context; inflammatory measures are non-specific and these domains must not replace validated cardiovascular models
Gastrointestinal and gut–brain phenotype	Stool form and frequency; abdominal pain or bloating; relevant gastrointestinal history; laxatives, antibiotics and bile acid-active drugs	Serum C4 and FGF19, targeted faecal or plasma bile acids and intestinal transit assessment [57–59]; standardised intestinal permeability assessments and exploratory autonomic or neuroimmune read-outs	Defines contextual bowel phenotypes and potential confounding; symptoms and advanced measures are biologically non-specific and are not validated cardiovascular predictors
Hepatobiliary phenotype	ALT/AST; ALP/GGT/bilirubin; clinically indicated ultrasound for hepatic steatosis, gallbladder abnormalities or biliary dilatation [116, 117]	Non-invasive fibrosis assessment, including elastography, and specialist imaging or hepatobiliary evaluation according to clinical indication	Identifies liver or biliary comorbidity and major confounding; does not directly measure bile acid synthesis, microbial transformation or receptor signalling
Microbiome–bile acid molecular phenotype	Standardised stool and blood collection with documentation of fasting state, sampling time, diet and medication exposure; no validated routine molecular panel	Targeted plasma and faecal bile acid species and ratios [57–59]; metagenomic or metatranscriptomic functional profiling [66, 67]; strain-level BSH isoenzyme and substrate specificity [103]; downstream bile acid-transforming capacity [104]; direct enzyme assays and multi-compartment profiling	Supports function-informed molecular interpretation; analytical thresholds, pipeline reproducibility, compartment concordance and incremental clinical value remain unestablished
Exposure and longitudinal response context	Fibre type and dose, polyphenol-rich foods and dietary fat quality [39, 51, 69, 88–90]; feeding–fasting timing, fermented foods and coffee [91–93]; medications, sleep, physical activity and adherence	Repeated digital exposure data synchronised with longitudinal microbiome, bile acid and clinical measurements	Helps interpret temporal variation, adherence and apparent intervention response; vulnerable to self-report error, reverse causation and residual confounding

Note. This proposed framework is intended for prospective evaluation and does not represent a validated cardiovascular screening, risk-prediction or treatment-selection pathway. Tier allocation may vary according to clinical indication, analytical availability and study setting. Abbreviations: *ALP* alkaline phosphatase, *ALT* alanine aminotransferase, *AST* aspartate aminotransferase, *BA* bile acid, *BSH* bile salt hydrolase, *C4* 7α-hydroxy-4-cholesten-3-one, *CVD* cardiovascular disease, *FGF19* fibroblast growth factor 19, *GGT* gamma-glutamyl transferase, *HbA1c* glycated haemoglobin, *PPPM* predictive, preventive and personalised medicine

Prospective evaluation of this predictive framework will require longitudinal metagenomic and metabolomic profiling with standardised, repeated sampling [119]. Single-cell, organoid and organ-on-chip systems may complement these studies by testing selected causal mechanisms, but they cannot substitute for clinical validation. Machine-learning approaches may be explored for multidomain risk stratification, but future model studies should be reported in accordance with TRIPOD + AI and appraised for quality, risk of bias and applicability using PROBAST + AI. At minimum, studies should report discrimination and calibration, use appropriate internal validation during model development, undertake external evaluation where feasible, and determine whether the model adds clinically meaningful value beyond established cardiovascular risk models [120, 121]. Mitochondrial biosensoric read-outs and endothelial function tests may be explored as complementary research measures of energetic or vascular stress, but their incremental predictive value within a microbiome–bile acid framework remains unvalidated [52, 122]. We therefore recommend prospective validation of multi-compartment bile acid, microbiome and functional biomarker panels before they are incorporated into routine cardiovascular risk assessment.

### Targeted prevention across disease stages

The preventive potential of the gut microbiota–bile acid axis reflects its biological plasticity, but the translational maturity of candidate interventions differs substantially. Established healthy dietary patterns, improvements in overall diet quality and adequate dietary fibre intake are clinically accessible components of cardiovascular prevention [69, 79, 88]. By contrast, microbiome-guided selection of prebiotics or probiotics, defined live biotherapeutics and bile acid-directed interventions remain investigational in cardiovascular PPPM [102, 105, 106, 109]. Future studies should prioritise interventions with a prespecified mechanistic target, standardised exposure and adherence assessment, and clinically relevant cardiovascular or cardiometabolic endpoints.

In secondary prevention and established cardiovascular disease, microbiota- or bile acid-directed approaches should currently be considered investigational adjuncts rather than established strategies for interrupting disease progression or heart–gut feedback loops. Candidate research approaches include defined live biotherapeutics, faecal microbiota transplantation, selected bile acids or receptor modulators, and repurposed pharmacological agents evaluated alongside guideline-directed cardiovascular therapy [109, 112]. These approaches require phenotype-stratified trials with prespecified microbial or bile acid targets, measures of functional response or engraftment where relevant, safety monitoring, and mechanistic as well as clinically meaningful cardiovascular endpoints. None should be regarded as routine cardiovascular treatment before efficacy, safety and incremental benefit over established care have been demonstrated.

### Personalisation of medical services

The rationale for investigating personalised intervention arises from the species-, compartment-, receptor- and disease-stage dependence of bile acid actions. Future studies should pre-specify the bile acid species and sampled compartment, the intended receptor or metabolic pathway, and the hypothesised direction of change, and should test whether this function-informed approach improves biological or clinical outcomes compared with non-stratified intervention. No such strategy has been validated for routine cardiovascular care. The same caution applies to live biotherapeutics: their effects are strain-specific, and prospective studies should determine whether matching defined functional properties to an individual’s microbial ecology, metabolic profile and clinical context improves outcomes [102].

Future studies should evaluate whether baseline multidomain phenotyping—including microbial functions, plasma or faecal bile acid profiles, bowel function, hepatobiliary findings, metabolic–inflammatory status, and diet and medication exposure—improves responder stratification for dietary, prebiotic, live-biotherapeutic or bile acid-directed interventions. Current evidence does not support routine use of these profiles to select patients for probiotics, faecal microbiota transplantation or bile acid-directed treatment. Proof-of-concept dietary studies suggest that temporal changes in the axis may track individual metabolic responses [86], while digital biomarkers may provide complementary information on diet, activity, sleep, adherence and blood pressure [111]. Adaptive, responder-stratified and n-of-1 designs could be used to evaluate this framework. For live-biotherapeutic studies, selection criteria should be prospectively tested against a prespecified functional deficit, the intended direction and anatomical compartment of bile acid change, and strain safety rather than taxonomy alone.

### Beyond the state of the art: toward cardiovascular 3PM

Previous reviews have summarised gut microbiota, bile acid metabolism or individual cardiovascular phenotypes largely from mechanistic or disease-specific perspectives. The added value of the present framework is to reorganise the gut microbiota–bile acid axis across the health–suboptimal health–disease continuum and to specify how this axis may contribute to predictive diagnostics, targeted prevention and personalisation of cardiovascular care. Rather than focusing on a single molecular target, this framework emphasises stage, compartment, receptor context, interindividual variability and dynamic monitoring.

This approach is consistent with the broader PPPM/3PM shift from reactive disease care to proactive, individualised health management [7]. However, it should be regarded as a translational framework requiring validation rather than as an already established clinical pathway. Moving the field forward will require longitudinal multi-omics, multi-compartment bile acid profiling, standardised analytical pipelines, human-relevant mechanistic models, digital health monitoring and responder-stratified clinical trials. If these requirements are met, the gut microbiota–bile acid axis may become a useful component of cardiovascular PPPM by helping to identify who is at risk, when to intervene, which node to target and how to personalise prevention or adjunctive treatment. Any future axis-guided model should be evaluated for incremental value over, rather than as a replacement for, established cardiovascular risk assessment and evidence-based preventive or therapeutic care.

## Data Availability

No datasets were generated or analysed during the current study.
